# An Integrated Recovery-oriented Model (IRM) for mental health services: evolution and challenges

**DOI:** 10.1186/s12888-016-1164-3

**Published:** 2017-01-17

**Authors:** Barry G. Frost, Srinivasan Tirupati, Suzanne Johnston, Megan Turrell, Terry J. Lewin, Ketrina A. Sly, Agatha M. Conrad

**Affiliations:** 1 School of Psychology, Faculty of Science and Technology, University of Newcastle, Callaghan, NSW 2308 Australia; 2 Centre for Brain and Mental Health Research, Hunter New England Mental Health and the University of Newcastle, Callaghan, NSW 2308 Australia; 3 Hunter New England Mental Health, Newcastle, NSW 2300 Australia; 4 School of Medicine and Public Health, Faculty of Health and Medicine, University of Newcastle, Callaghan, NSW 2308 Australia

**Keywords:** Evidence-based psychosocial interventions, Hope, Mental health services, Models, Recovery, Recovery-oriented, Rehabilitation, Serious mental illness

## Abstract

**Background:**

Over past decades, improvements in longer-term clinical and personal outcomes for individuals experiencing serious mental illness (SMI) have been moderate, although recovery has clearly been shown to be possible. Recovery experiences are inherently personal, and recovery can be complex and non-linear; however, there are a broad range of potential recovery contexts and contributors, both non-professional and professional. Ongoing refinement of recovery-oriented models for mental health (MH) services needs to be fostered.

**Discussion:**

This descriptive paper outlines a service-wide Integrated Recovery-oriented Model (IRM) for MH services, designed to enhance personally valued health, wellbeing and social inclusion outcomes by increasing access to evidenced-based psychosocial interventions (EBIs) within a service context that supports recovery as both a process and an outcome. Evolution of the IRM is characterised as a series of five broad challenges, which draw together: relevant recovery perspectives; overall service delivery frameworks; psychiatric and psychosocial rehabilitation approaches and literature; our own clinical and service delivery experience; and implementation, evaluation and review strategies. The model revolves around the person's changing recovery needs, focusing on underlying processes and the service frameworks to support and reinforce hope as a primary catalyst for symptomatic and functional recovery. Within the IRM, clinical rehabilitation (CR) practices, processes and partnerships facilitate access to psychosocial EBIs to promote hope, recovery, self-agency and social inclusion. Core IRM components are detailed (*remediation* of functioning; collaborative *restoration* of skills and competencies; and active community *reconnection*), together with associated phases, processes, evaluation strategies, and an illustrative IRM scenario. The achievement of these goals requires ongoing collaboration with community organisations.

**Conclusions:**

Improved outcomes are achievable for people with a SMI. It is anticipated that the IRM will afford MH services an opportunity to validate hope, as a critical element for people with SMI in assuming responsibility and developing skills in self-agency and advocacy. Strengthening recovery-oriented practices and policies within MH services needs to occur in tandem with wide-ranging service evaluation strategies.

**Electronic supplementary material:**

The online version of this article (doi:10.1186/s12888-016-1164-3) contains supplementary material, which is available to authorized users.

## Background

Disorders such as schizophrenia were historically viewed as chronic, degenerative illnesses, with little prospect of improvement or recovery. These negative and debilitating notions of serious mental illness (SMI) were challenged by the consumer movement, with recovery perspectives bringing a new sense of meaning and purpose to individual’s lives, even though symptoms might remain [1–4]. However, in the absence of clear operational or scientific definitions of ‘recovery’, it was questioned whether the process would be understood and amenable to collaborative interventions [5], or the value of the term compromised [6] and potentially commandeered by those seeking to reduce service costs [7, 8]. Concerns that recovery-focused initiatives could default to rhetoric rather than practice were also raised [9, 10].

Consumer research identified recovery as both a process and an outcome, involving factors related to personal wellbeing and social inclusion, which were distinct from traditional clinical domains [4, 11]. Nevertheless, some scepticism remains around the notion of recovery [12], coupled with concerns that the burden of risk will be borne by families and carers [13]. It is generally accepted that improved mental health (MH) outcomes can be achieved through access to a range of psychosocial evidence-based interventions (EBIs) [10, 14–16]. However, sufficient service ‘infrastructure’ needs to be activated to ensure recovery-oriented approaches are successfully embedded into everyday practice and access to EBIs is enhanced.

Advances in psychopharmacology made it possible for many people with SMI to be discharged from long-stay care. However, they were often discharged from highly structured inpatient environments with little provisioning for their needs, which according to some reports, did not extend beyond a prescription [17]. It became increasingly apparent that many individuals experience a constellation of signs and symptoms superimposed and interacting with a background level of impairment and disability. Function is often impaired across multiple domains (e.g., cognition, living skills, social skills, occupation/education) and the level of impairment can often be exacerbated by relapse and deteriorate further with subsequent episodes [15].

Whilst psychopharmacological treatments have improved and are considered fundamental to illness management, their role in the restoration of skills considered essential for a satisfying and fulfilling life is at best limited. For example, Meltzer [18] was unable to identify a correlation between amelioration of positive symptoms and social outcomes. It is also evident that medications have not solved the problem of relapse [19] and carry significant side effects and risks, including over-reliance, poly-pharmacy and inappropriate use [20]. Following a 20 year longitudinal study, Harrow et al. [21] state that “*antipsychotics are not effective in eliminating or reducing psychosis for the great majority … and may impede recovery of some …*” (p. 3013). Further, Deacon [22] suggests that under biomedical treatment models there has been a sharp increase in psychiatric medication use, a broad lack of clinical innovation, and poor MH outcomes.

Despite calls for reform, the disparity between the recovery needs of individuals with SMI and service delivery paradigms is reflected at several levels. For example, among young people, schizophrenia remains one of the top ten causes of disability [23]. People with psychotic disorders represent 25% of total disease burden [24] and schizophrenia is the 3rd most important disease in terms of years lived with disability for those aged 15–44 years [25].

Poor physical health is also experienced by many people with psychotic disorders, with 45.1% classified as obese and 33.5% assessed as having low physical activity [26]. The majority of people with SMI are also unemployed (78.5%), have poor education levels, impaired social skills (63%), and limited contacts [26]. Consequently, the estimated annual economic cost in Australia for all psychotic disorders is $4.91 billion from a societal perspective and $3.52 billion from a government perspective [27]. Moreover, even though annual costs have been relatively stable (over the 2000–2010 decade), there has been a significant redistribution of costs to the non-health sector, in line with Australian government initiatives [28]. The high and continuing levels of burden associated with SMI have prompted some authors to call for ‘widespread systemic change’ to MH systems, promoting an increased emphasis on shared decision making, independence (e.g., financial, residential, personal) and social connectedness [10].

### Regional opportunities and imperatives

Like many countries, Australian MH services are currently in a state of transition, including: formulation of national frameworks with an increased focus on recover-oriented care provision [29–31]; development of a new Australian MH Care Classification [32]; and introduction of Activity Based Funding [33]. In broad terms, **recovery-oriented service delivery**: “*… is centred on and adapts to people’s aspirations and needs, rather than people having to adapt to the requirements and priorities of services*” and it has a “…*responsibility to provide evidence-informed treatment, therapy, rehabilitation and psychosocial support that assist in achieving the best outcomes for people’s mental health, physical health and wellbeing*” [30], p. 26.

Within New South Wales (NSW), planning commenced in 2005 to establish a number of sub-acute inpatient MH units, with the primary goal of improving access for people with SMI to recovery-focused rehabilitation services that were highly integrated and rigorously evaluated [34]. This provided an opportunity for Hunter New England Mental Health services to develop an innovative model of care at a level of service delivery that had not previously been explored. Details about the specific 20-bed, sub-acute Intermediate Stay Mental Health Unit (ISMHU) that was initially established are provided elsewhere, together with our preliminary service evaluation [35].

Importantly, development of a new level of regional MH care necessitated consideration of all of the potential MH service pathways and partnerships, together with their treatment models and intended goals. Within this context, and given the limited availability of established service-wide, recovery-focused models of care [36–39], a broader framework for an Integrated Recovery-oriented Model (IRM) for MH services was formulated, which sought to support and promote ‘***remediation***, ***restoration*** and ***reconnection***’.

The primary purpose of this paper is to outline the IRM and to stimulate ongoing refinement of recovery-oriented service models. Evolution of the IRM is characterised with respect to five broad challenges. The first three challenges relate to identification of: 1) relevant recovery perspectives; 2) overall service delivery frameworks and models; and 3) key features and processes associated with current specialised clinical rehabilitation (CR) interventions for people with enduring SMI. The fourth, or central challenge, is to draw together the main elements from these first three challenges into a coherent, service-wide IRM for MH service delivery. The fifth challenge relates to devising relevant implementation, evaluation and review strategies for recovery-oriented MH service models and components.

### Recovery perspectives

#### Challenge 1

Identifying the aspects of personal and clinical ‘recovery’ and related approaches that need to be considered in re-designing ‘recovery-oriented’ MH services.

#### Recovery possibilities and needs

Research has shown that recovery is possible [40, 41] and that people with SMI value the opportunity to participate and contribute to society [42]. However, for many there is limited access to EBIs that may prove effective in supporting hope and restoring confidence and competence [43]. Mojtabai et al. [44] found that more than 50% of people with schizophrenia received either no treatment or suboptimal treatment. Torres-González et al. [45] identified six areas of specific need: frequent complications and co-morbidities (e.g., substance misuse); psychological, social and economic needs; early interventions to reduce illness progression; treatment augmentation with rehabilitation EBIs; maintenance of service contacts; and greater research efforts into existential needs. Better access to psychosocial interventions and well-managed medication are warranted [14, 45], together with a shift away from case/risk management practices to service models that facilitate access to EBIs [10, 20].

#### Recovery goals

The term recovery is clearly multi-layered. Nevertheless, it carries an unequivocal message of a better outcome, conveying a sense of hope; it may also carry expectations in regard to interventions, timeframes and supports. Attempts to reintroduce hope and optimism are based on the view that recovery is possible even though residual limitations may remain. Unlike physical medicine, where recovery goals are generally well understood, the role and significance of rehabilitation for people with SMI has been less well understood - even though psychiatric rehabilitation has always been about ‘recovery’ [19] and supporting self-determination and independence through improvements in wellbeing and role functioning.

Snyder et al. [46] described hope as “*the person’s perceived ability or internalised belief that he or she can produce goals, pathways and agency*” (p. 89), suggesting that, as a goal directed motivational process, hope requires constant feedback and agency. If hope is a catalyst for change and improved health outcomes [46], the question arises as to how hope is both generated and sustained. This also brings into focus the ethical requirements of *beneficence* (doing good) and *maleficence* (avoiding harm) that typically guide health service provision. Some recovery-oriented frameworks propose that hope may be generated through service and cultural reforms; for example, *“… the physical, social and cultural service environment inspires hope, optimism and humanistic practices for all who participate in service provision*” ([47], p. 7). Although such statements are very positive, they run the risk of being overpowered and reverting to rhetoric, unless driven by outcomes that reconfirm the considerable investments in recovery.

Le Boutillier et al. [37] suggested that promoting citizenship and a clear sense of place are core goals for recovery-oriented MH services, the primary purpose of which is to encourage self-agency. Validating personal goals can also help to reduce a client’s sense of frailty and hopelessness. Liberman and Kopelowicz [5] proposed that as improvements are made in a range of personally valued domains, more subjective qualities such as hope, empowerment and autonomy become evident. Snyder et al. [46] suggested that the processes of hope and rehabilitation “*fuel each other in an iterative manner over the temporal course of treatments*” (p. 107). Recovery can be complex and non-linear, with hope seen as critical in shaping and sustaining improvements in a range of skill domains, consistent with social inclusion [48, 49].

#### Recovery processes

Early access to rehabilitation interventions has been associated with better functional outcomes [50]. Making rehabilitation available across the continuum of care may reduce health costs by shortening hospital admissions, reducing activity limitations, and improving quality of life. More generally, the discipline of psychiatric rehabilitation has contributed much to improving service delivery and outcomes [17]. Psychiatric rehabilitation challenged the MH system to think more expansively and respectfully about people with SMI, promoting choice, shared decision-making, consumer involvement, and a focus on inherent strengths and recovery possibilities.

The discipline of psychiatric rehabilitation promoted the adoption of a broad, holistic approach and advocated for access to quality residential, education and employment opportunities. Quality frameworks were also introduced, including comprehensive multidisciplinary and inter-service team reviews. Due to the obvious synergies with the recovery approach, rehabilitation services have been proactive in adopting consumer oriented recovery strategies. Much has also been done to reduce the negative approach associated with the official nosology of schizophrenia, in which therapeutic nihilism and stigma have operated as self-fulfilling prophecies [5]. Perhaps, reluctance to accept the discipline stems from the fact that psychiatric rehabilitation is relatively easy to define but, as highlighted by Anthony and Farkas [17], any explanation belies the complexities of the processes involved.

An understanding of personal recovery as a *subjective experience* has emerged and this meaning now underpins MH policy internationally e.g., [38, 51]. While the provision of recovery-oriented care is a guiding principle, implementing recovery-oriented or recovery-enabling [52] practices requires transformations within MH systems [10, 38, 39]. In some sectors, such as MH inpatient settings, there is limited research directly addressing recovery-oriented practice [39, 53]. However, a recovery enabling framework has been proposed to address workforce gaps in core recovery competencies among inpatient providers [52].

Until recently, the focus was almost exclusively on clinical recovery [54]. Central to the delivery of recovery-oriented services is a shared understanding of recovery between consumers, carers and health professionals [51]. Recovery-oriented psychiatric rehabilitation can be seen as supporting people with SMI in the pursuit of a meaningful life [55]. As recovery is an ongoing and non-linear process, recovery-oriented experiences and opportunities during periods of hospitalisation also need to be adequately addressed [52].

#### Recovery contexts

Once again, it needs to be explicitly acknowledged that recovery experiences, opportunities, trajectories, and evaluations are inherently personal. Among people with SMI, recovery is generally viewed as “*a journey of small steps*”, within which participation in everyday activities is “*frequently considered as both facilitators and indicators of recovery*” ([10], p. 237). Moreover, while the current paper is primarily about recovery-oriented MH service provision, there are a broad range of potential recovery contexts and contributors and, for many people, professional interventions may play a relatively minor or time-limited role [56]. On the other hand, individuals with enduring SMI are likely to be influenced proportionately more by the attitudes and practices of specialised MH, general health, and community managed services. Importantly, key processes associated with recovery (e.g., sustaining hope, promoting self-agency and reconnection) need to occur both within and outside of MH services [56] and, where possible, be enhanced by integrated, recovery-oriented practices.

### Service delivery frameworks and models

#### Challenge 2

Reconciling the broad array of general and specialised service delivery frameworks, models and intervention strategies of potential relevance to ‘recovery-oriented’ MH services.

There are numerous recommendations about service delivery approaches, ranging from general health or MH focused over-arching ‘frameworks’, through broad ‘intervention strategies’ or ‘models’, to specific ‘targeted interventions’. The WHO International Classification of Functioning, Disability and Health [ICF, 57] provides a general framework for considering the spectrum of needs of people with SMI. Integrating medical and social models for people with health conditions, the ICF focuses on human functioning, activity and participation, rather than disease and disability. It also provides a comprehensive guide to the identification of a range of protective and risk factors. For example, at the level of body function, the ICF framework includes consideration of psychotic symptoms, poor concentration and memory, low self-esteem and confidence. Activity limitations may include poor self-care, poor physical health, social withdrawal, and an inability to follow instructions. Participation restrictions may be reflected as the inability to continue education, difficulties maintaining social relations, problems with accommodation and accessing recreational activities. Consequently, an array of recovery-oriented approaches may be required to promote and sustain hope and resilience, facilitating improvements in personal functioning, activity and social participation.

The ICF has previously been implemented in an Italian psychiatric rehabilitation setting and reported to be a helpful framework among people with SMI, promoting a common language and integrated treatment model, supporting the development of client focused individual rehabilitation plans and improving services [57]. Similarly, individualised approaches to recovery in vocational rehabilitation have found positive effects on both clinical and employment outcomes [58]. Although the research literature provides some assistance in regard to recovery-oriented frameworks, it provides limited guidance on optimal delivery systems or recovery-oriented models for MH services.

Perkins and Slade ([59], p. 33) noted that “*there can be no ‘blueprint’ for recovery – each person must find their own way*”, although key factors important in supporting recovery-oriented practice and transforming MH services have been identified in the recovery literature. Le Boutillier et al. [37] proposed a conceptual framework to guide practice, focusing on four domains: promoting citizenship; organisational commitment; supporting personally defined recovery; and working relationships. Hopper [60] viewed recovery as a therapeutic endeavour and proposed four stages in the recovery process: renewing a sense of possibility; regaining competencies; reconnecting and finding a place in society; and reconciliation. Rodgers et al. [61] employed a staged approach, mapping EBIs for each stage of the recovery process.

From a service model perspective, Thornicroft and Tansella [62] suggested service configurations should be balanced between hospital and community services, outlining three levels of care: primary care with specialist back-up; mainstream MH care; and specialised MH services. Specialised services included: early intervention; assertive treatment teams; alternatives to acute inpatient care; residential care and vocational rehabilitation. Adopting a slightly different approach, Flannery et al. [63] developed a service model based on the core functions required for a recovery-focused MH system: acute care (community teams and alternatives to inpatient care); emergency services; continuing care partnerships (assertive treatment teams, supported accommodation, therapy services, vocational rehabilitation and drop-in centres); and early intervention services. Although this pragmatic approach could be introduced with minimal cost, it is unclear how access to EBIs and other major requirements of recovery-focused models would be achieved. The fundamental tenant of any reform should be that recovery is supported as both a process and an outcome. If this does not occur, there is an inherent risk that traditional imperatives will prevail and re-establish a disconnected dichotomous system (e.g., acute/emergency vs. disability support services).

Slade et al. [38] identified ten validated interventions that support recovery by targeting key processes (connectedness, hope, identity, meaning and empowerment [CHIME]) [64], illustrative of the types of interventions expected in recovery-oriented MH systems. These included: peer support workers; advance directives (if future capacity is lost); wellness recovery action planning (WRAP) tools and processes [65]; illness management and recovery (IMR) [66]; the REFOCUS model (recovery-promoting relationships and work practices) [67, 68]; strengths-based models [69]; recovery colleges or recovery education programs; individual placement and support (IPS) [70]; supported housing; and MH trialogues (community forums). Many of these EBIs can be implemented regardless of the specific recovery-oriented model; although some have been evaluated predominantly in community MH settings [38]. Others involve more complex manualised pro-recovery interventions or modules, such as the REFOCUS model, IMR program, and WRAP, which also emphasises peer support in the development of individual recovery plans [65]. Strengths-based case management models supporting consumer directed care have also been implemented in both acute and community MH settings [69, 71], focusing on personal strengths and goals rather than deficits, and integrating a variety of EBIs. While all approaches support recovery, few provide an overarching framework and service-wide model for MH care provision.

Internationally, implementing recovery-oriented practices has posed challenges for MH services [65, 72]. In Australia, a need for MH systems transformation has also been identified, in order to provide a continuous recovery-oriented care framework that links acute inpatient and community services [73]. Recent conceptualisations of recovery-oriented practice have focused primarily on clinical and personal recovery; however, a new concept of service-defined recovery is seen as translating recovery into practice according to the goals and needs of an organisation [74]. This accords with earlier suggestions that an ideal model should “*link the abstract concepts that define recovery with specific strategies, that systems, agencies and individuals can use to facilitate it*” ([75], p. 482). While service approaches operationalising recovery-oriented practice are yet to be extensively evaluated, research on staff perspectives has identified perceived barriers (e.g., competing priorities in providing recovery-oriented support), which also highlight the need for a whole-systems approach in transforming services [74, 76].

### Clinical Rehabilitation (CR) within MH services

#### Challenge 3

Building on the core elements of psychosocial and MH rehabilitation, to facilitate service provision along a recovery-oriented continuum, with specialised clinical rehabilitation processes and services nearer to one extremity, delivering targeted MH interventions and supporting people with enduring SMI.

In part, we use the expression ‘CR within MH services’ to draw a distinction with ‘disability support’ (associated primarily with care linked to enduring functional *impairment* or other activity *limitations*) and to de-emphasise the discipline-specific aspects of ‘psychiatric rehabilitation’, in favour of a recovery-oriented care continuum of relevance to all MH workers. All of these approaches have roles to play but require different skills sets, competencies and professional and clinical processes. Encouraging clients to progressively assume independence and responsibility for their own care is axiomatic to CR and consistent with personal recovery approaches [6, 16, 17, 19, 77]. Given that CR provides a unique opportunity to empower people with SMI to assume greater levels of self-agency, the question arises as to how these opportunities can be further realised within service delivery models that not only respect this role but also complement and enhance opportunities for recovery and social inclusion?

CR employs a set of interventions and processes that aim to achieve and maintain optimal functioning in the client’s environment of choice. CR is about helping individuals to realise their personal goals, in a supportive context that builds trust and confidence in self-agency. It is about affirming and reaffirming that the investment of hope in personal coping and everyday functional skills has been justified and, in so doing, support the independent exploration of new and more satisfying personal goals.

Developing interventions and supports that promote recovery and challenge commonly held stereotypes, which by definition disable and segregate, is a complex undertaking. Hope is a key factor in this process and, in taking the first tentative steps to regaining a sense of control and self-agency, it is vitally important to understand the risks involved and to ensure trust and personal dignity are protected. Ensuring that an individual’s investment in rehabilitation and recovery processes is supported, and not adversely affected as new goals are explored, is also critical.

Depending on individual recovery goals, CR may involve single or multiple EBIs delivered by a skilled practitioner, in conjunction with a CR team. The interventions should be developed in a collaborative, empowering and optimistic manner, based on a thorough understanding of the person’s goals and abilities (including both strengths and vulnerabilities). The plan may also be cross-sectoral, involving health professionals working in conjunction with general practitioners (GPs), community support agencies, as well as educational, employment and housing organisations. From a service-led recovery perspective [74], it should also be recognised that there may need to be different service streams even within specialised CR services, reflective of variations in the complexity of client needs and available resources; for example, some service streams may offer targeted, time limited EBIs, while others provide more of a ‘continued care’ approach, supporting clients with enduring SMI to maintain their MH and community tenure.

#### CR principles and priorities

Foremost among the key features of CR are the principles that guide the delivery of recovery-focused interventions: recovery-oriented; promoting independence; person-centred; flexible, responsive and inclusive; accommodating different learning styles; focusing on strengths; utilising EBIs; providing integrated multidisciplinary care (including service continuity); and facilitating community and environmental supports. Some of the CR processes that flow from these principles are detailed in Table 1, including establishing recovery-oriented goals, undertaking assessments and recovery planning, delivering interventions, and clinical review or recovery-focused tracking.

**Table 1:** Clinical rehabilitation (CR) processes

*Planning and Diagnosis*		*Intervention and Review*		*Transfer of Care/Discharge*	
Recovery Goals	CR Assessment	Recovery Planning	CR Interventions	Clinical Review or Recovery-focused Tracking	
**Aspirations** - Hope of a better life may include: wellness enjoyment, participation, contribution and opportunity. **Personal** - working with an individual’s goals no matter how well grounded, is pivotal in fostering commitment to recovery processes. **Self-identified** – imposing goals that are incongruent with the individual’s is simply counter-productive and diametrically opposed to the tenants of CR. **Well formulated** - using assessment tools^a^ that have credibility with a person may assist in discussing and formulating recovery goals.	**Comprehensive** – thorough and holistic, not adopting a pathological view of SMI, but unashamedly a comprehensive appraisal of relevant factors to assist in the formulation of a collaborative recovery plan. **Multiple domains** - may include: medication, treatment, co-morbidity, substance-use, physical & cognitive issues, coping, daily living skills, living arrangements, education & employment, family interactions, social, sexual and existential needs & stage of change. **Function oriented** – may include an array of issues in domains of functioning, activity & participation, role & impact on environmental and personal factors. **Promoting hope** – the knowledge gleaned assists clinicians to work effectively with clients & their family in generating and validating hope.	**Collaborative** – may be developed using tools such as the MHRS^a^. Recovery-oriented plan outlines individual recovery needs and develops strategies dependent on motivation for change in specific domains. **Evidence-based -** guiding access to a range of interventions (e.g., cognitive remediation, skills training, family interventions, employment & education strategies), as well as support & environmental adaptation. **Delivery methods** - interventions may be detailed as concurrent, sequential, in individual or group settings, as well as identifying who participates (family, carers, friends, support workers). **Coordinated** – across clinical and non-clinical interfaces, as well as addressing the interaction of CR & pharmacological intervention.	**Goal focused** - related to a range of personal, social & environmental factors, not diagnosis dependent. Interventions assist in achieving goals & improving mental & physical health. **Individually tailored** –interventions are individually tailored but, to assist clinicians in recovery-oriented service provision, core interventions may be linked with domains of recovery (e.g., using the MHRS^a^). **Integrated programs** – provide a foundation for developing strategies and interventions. Core & elective programs operate in individual, group, milieu/residential and community settings. **Structured** – collaborative, goal focused, evidence-based and motivational to promote generalisation, and accommodate different learning styles and abilities. To support achievement, higher level therapy programs are run in parallel to compliment activity-based programs.	**Aim** to ensure the highest level of care & that: 1. Strategies are comprehensive, responsive & positive; 2. Support is available to the CR clinician at every step; 3. Continuity maintained through information sharing; 4. Concurrent interventions are implemented and monitored; 5. Early intervention strategies are available; 6. Multidisciplinary team skills are available; 7. Risks are quickly identified & resolved; 8. Interventions are evidence &/or practice-based; 9. Positive recovery-oriented outcomes are shared; 10. Care coordination facilitates high quality service; 11. Opportunities exist to build service networks and linkages; 12. Key performance indicators are discussed and reviewed. The review provides a forum to oversee, support & co-ordinate service delivery & maximise recovery possibilities.	
				**Time demands** - time intensive due to complexity of issues & need to ensure a positive and productive milieu. Recovery trajectories are complex & dynamic. Work contexts can be demanding, particularly when the time course is extensive & progress minimal.	**Processes** – innovative, recovery-focused, inclusive & holistic. Review should be led by a senior CR clinician due to complex processes & time-frames, & include client, family, peer-support & relevant agencies. Feedback informs goal development & collaborative interventions.
				**Recovery pathways** - may be simple & linear or interspersed with minor setbacks, even relapse. Incremental recovery in some domains & profound in others. Gains may be interdependent. Numerous reiterations of CR processes may be required to trigger a decision to adopt a more inclusive & adaptive approach (e.g., for SMI & substance misuse).	**Achievements** – Clinical review can objectively affirm achievements; facilitate development of options &/or determine when another strategy is warranted; & aid making complex decisions regarding level of service need. Achievement of self-determination in several domains may require minimal short-term interventions accompanied by follow-up & review.

^a^For example, collaborative measures such as the MHRS provide a framework and shared language for discussing pathways to recovery and wellbeing that may be employed across a range of service settings including clinical and non-clinical. The strength of this particular tool lies in its ability to connect with people with SMI in identifying need, developing individually tailored recovery and relapse prevention plans, and reflecting progress along the recovery journey

People change and grow, and various factors promote positive adaptation, such as setting your own goals, learning new skills, hope, and self-efficacy [17]. With respect to specific or targeted CR intervention priorities, Mueser et al. [16] recently classified psychosocial interventions according to whether the evidence was sufficient or promising. Included among the established EBIs were: cognitive behavioural therapy for psychosis; cognitive remediation; family psycho-education; illness self-management training; social skills training; and supported employment. Other interventions considered to be very promising [16] were: social cognitive remediation [78]; cognitive adaptive training [79]; integrated psychological therapy [80]; healthy lifestyle interventions [81]; and supported education [82]. Additional interventions with an evidence base included: motivational interviewing reviewed by [83, 84]; errorless learning [85]; skill building reviewed by [86]; and family interventions reviewed by [87].

Specialised CR services may also require a staffing compliment and roster arrangements that depart from traditional approaches. Ideally, staff should be recruited against a set of values and competencies consistent with rehabilitation and recovery-oriented approaches, including: openness; empathy and encouragement; supporting responsible risk taking; a positive outlook; a collaborative focus on client’s inner resources and strengths, and a preparedness to go the extra distance [88]. Experience suggests that CR staff also need to be patient, resourceful, and innovative, and enjoy problem solving. Professional background and training is also important, as some professions have extensive theoretical and practical training in provision of complex interventions. For example, increasing the number of occupational therapists, social workers and psychologists, relative to those with generalist training, may significantly increase service capacity and recovery focus. However, such guidelines may be misleading, as some generalist-trained staff with a passion for CR may make outstanding contributions. Importantly, CR teams should also include consumer advocates, as these staff may provide direct assistance to clients and clinical staff, and help ensure that the team retains a strong client-centred recovery-oriented approach.

### Integrated Recovery-oriented Model (IRM)

#### Challenge 4

Developing a recovery-oriented model for MH service delivery (promoting ‘remediation, restoration and reconnection’) that provides both an overarching, inherently collaborative and integrated approach, together with identification of opportunities for targeted specialist CR initiatives.

The IRM was designed to support the recovery needs of people with SMI by improving access to a range of EBIs provided within a service context that reinstates hope, rebuilds competencies and provides opportunities to reconnect. Three foundation elements or functions of this service model that partner with the individual client include: acute/emergency MH care; specialised CR; and community managed/non-government organisations (CMOs/NGOs) providing community integration services*.*

The IRM operates as a tripartite agreement, with each of the partners providing recovery-focused services in an integrated and seamless manner. Each of the core services may also operate in conjunction with a range of other specialist services (e.g., sub-acute inpatient, substance misuse, neuropsychiatry) and community-based organisations, including GPs, accommodation services, employment services, education providers, drop-in centres, community participation and recreation services. To ensure continuity, the IRM requires flexibility, transparency and responsiveness, but with the degree of service involvement titrated according to client recovery needs. Clearly, this requires a solid understanding by all partners of service and management core functions and processes. Consequently, a major strength of the IRM is the ability to safeguard hope and self-esteem by intervening early to preserve coping and functional skills across a number of domains, including everyday living skills, accommodation, social networks, employment and education endeavours.

Key principles guiding service delivery within the IRM include: 1) services are recovery-oriented; 2) care delivered is person-centred, holistic and inclusive; 3) care enables and supports choice and self-management; 4) services are integrated across the care continuum; 5) service delivery is seamless and complementary across all providers (i.e., no ‘wrong door’); 6) services and care are based on the most appropriate available evidence; 7) partnerships with other services, government departments and CMOs/NGOs are integral to service delivery; 8) consideration of equity issues informs decisions about services and care; 9) information technologies are used to improve access to care, facilitate enhanced collaboration and communication within the service, consumers, their families and carers; and 10) services and care delivery is aligned with national, state and local directions.

**Fig. 1:**
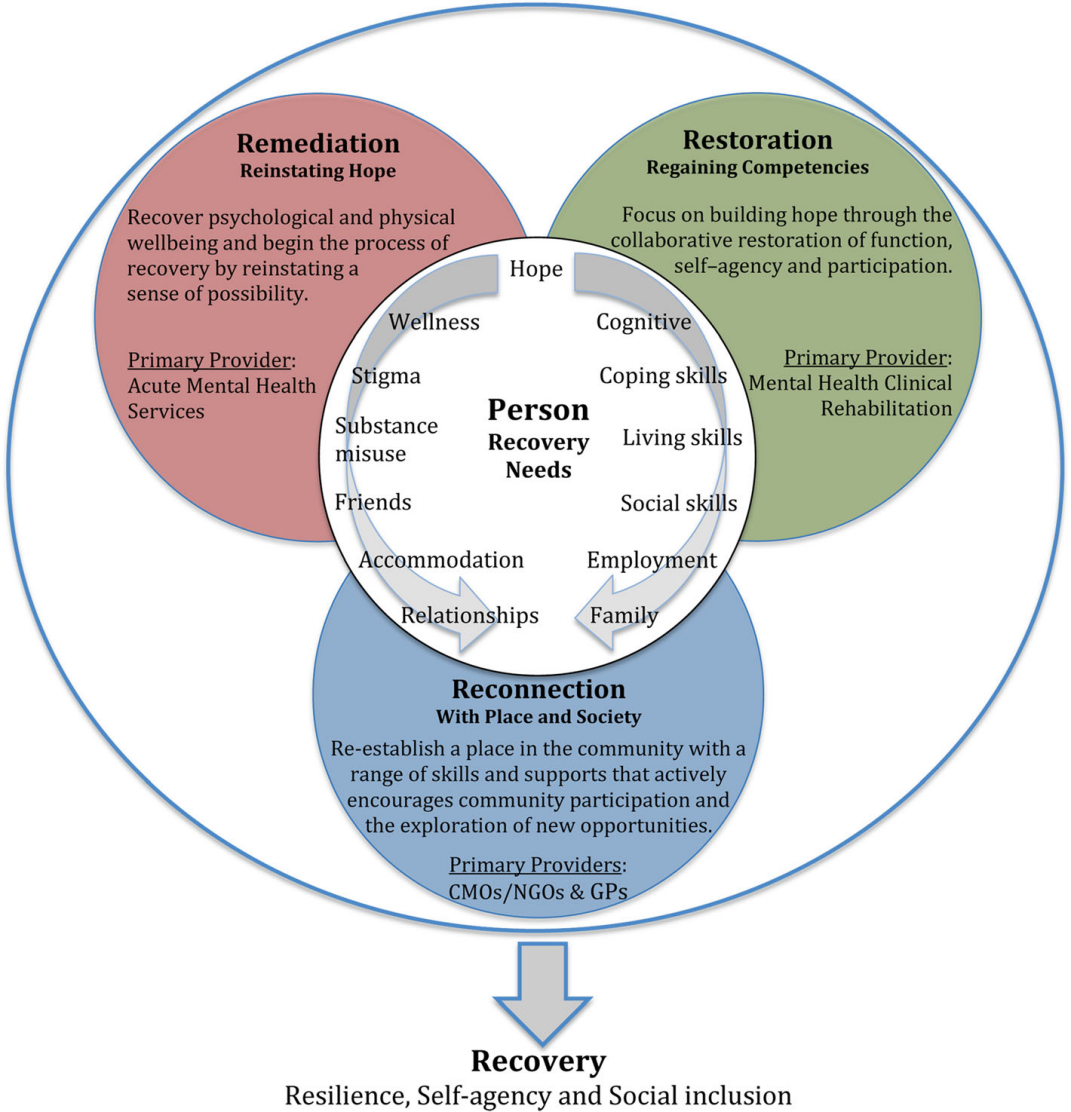
Integrated Recovery-oriented Model (IRM) for mental health services

**Fig. 2:**
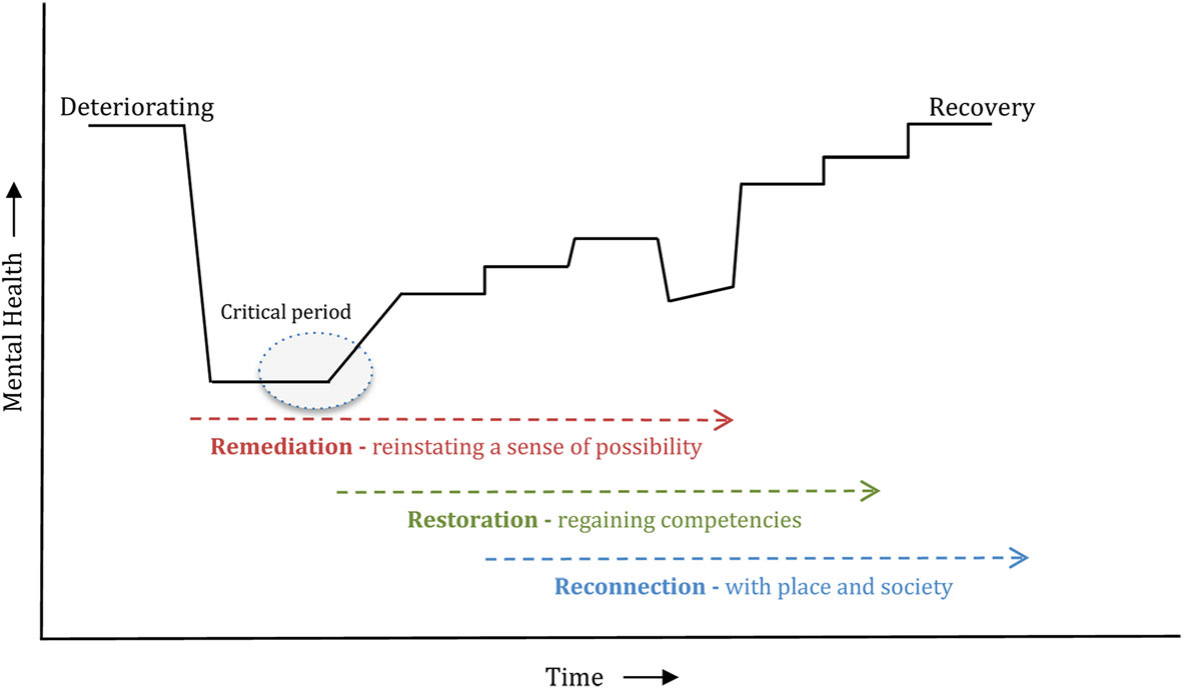
Integrated Recovery-oriented Model (IRM) - Phases of recovery

The three main components of the IRM have been based on the ICF concepts of function, activity and participation [89], but also incorporate elements identified by Hopper [60]. Under the IRM, it is proposed that acute services should focus on ameliorating positive symptoms and reinstating a sense of possibility. At the earliest available opportunity, CR services, supported by CMOs/NGOs, would begin to restore hope through the development of a range of skills pertinent to personal goals. As the client regains confidence, CMO/NGO services would focus on exploring opportunities that would reinforce personal recovery and reconnection with the community. However, it also needs to be acknowledged that there is variation across Australian States in the service delivery roles performed by CMO/NGO services, and even more so from an international perspective. The manner in which these remediation, restoration and reconnection components revolve around the person's changing recovery needs is highlighted in Fig. 1. The overlapping and, somewhat idealised, sequential phases of recovery are further illustrated in Fig. 2; acknowledging again that recovery can be multi-layered and non-linear [48, 49]. More detail about the complementary roles of the respective IRM components is provided below.

1. Remediation of functioning - reinstating a sense of possibility
  This phase is the start of a complex journey in which the key elements that generate and sustain hope must be carefully reintroduced and nurtured. The goals are to intervene early to reduce the psychological and social sequelae associated with the onset of illness. Building trust and hope that is real and sustainable will be critical in developing a positive adjustment to the diagnosis. This phase also provides an opportunity to address physical health issues, ensure safety, manage any legal and financial issues, and to identify other likely impacts on the person, their partners, families and friends. When a person’s coping and protective strategies have been breached, resulting in acute psychosis, they are likely to feel overwhelmed, shocked, confused, fearful, anxious, in denial and exhausted. These reactions may be fuelled by stigma and run the risk of being exacerbated by treatment and management plans that are: circumspect in their vision; fail to respect and value the person, their family’s needs and aspirations; or lack credibility in terms of delivery and coordination.
  Initial treatment provides an invaluable opportunity to reduce fear associated with the onset of symptoms and the diagnosis, and to commence development of a collaborative recovery-oriented plan that is consonant with the wishes and aspirations of the person and their family. To ensure that the client’s investment of hope is well placed, it is essential that there is a full understanding of their strengths, protective factors and possible risks. As with physical rehabilitation, care needs to be exercised as the events and triggers that precipitated the relapse are brought into sharp focus by an approaching discharge. The need for care is also reinforced by the knowledge that a successful resolution of positive symptoms does not necessarily indicate a return to pre-episode functioning. A thorough assessment is required to develop a supportive, individually tailored, multi-modal skill building program, which may be provided in combination with other treatments; a point highlighted in a recent review by Lyman et al. [86].
  The need for a holistic plan, which supports hope through a range of strategies that build confidence and competencies and addresses vulnerabilities, underscores the importance of the early involvement of rehabilitation specialists. While this phase will generally be led by acute MH services (which have specific expertise in treating positive symptoms), they also require the support of CR, and CMO/NGO services, in building confidence and hope in a plan that extends beyond the acute setting. In order to demonstrate an unequivocal commitment to the goals of the collaborative recovery-oriented plan, a number of relevant clinical and nonclinical services may need to be involved, including: emergency assessment and triage; acute inpatient and community services; community MH teams; early intervention programs; specialist clinicians; and associated links with GPs, sub-acute inpatient and other specialist agencies.
2. Restoration – enabling, regaining competencies
  The goal of this phase is to demonstrate that hope and the sense of possibility are valid constructs in the pathway to recovery. At the earliest opportunity, a range of EBIs should be available to assist in rebuilding or confirming personal, interpersonal and daily coping skills and competencies. This may also provide an opportunity to redress developmental gaps and lifetime goals, both of which could contribute to a renewed sense of self. As confidence is developed in personal coping skills and environmental adaptations, a more robust foundation for further pathway or goal-directed thinking should emerge. Exploring new and confirmatory experiences will obviously entail a degree of positive risk taking and comprehensive strategies may need to be in place to safeguard personal dignity. Throughout this phase, the focus will be unequivocally on the development of self-agency, particularly as it relates to mental and physical recovery, and social inclusion.
  For some people with SMI, the recovery journey may initially hold few protective factors and pose considerable challenges and risks. For example, a move from a highly structured inpatient unit to a loosely structured home or residential setting, with a questionable and fragile confidence in coping skills and supports, may pose major risks. Insufficient supports during this challenging period may propel a person to find membership in segregated company or attempting to self-manage through the use of non-prescribed substances. Transitional arrangements may provide an opportunity to build confidence and minimise stress, as well as providing a positive foundation on which to build essential psychological and everyday functional skills. The development of additional competencies may include: strategies to manage residual symptoms; cognitive skills; social skills; activities of daily living; physical health; family education and support; and supported education or employment. These interventions should be based on a comprehensive assessment, including usage of collaborative tools such as the Mental Health Recovery Star MHRS; [90], and a collaboratively developed recovery-oriented plan.
  CR services need to work in partnership with acute services, both inpatient and community, and CMOs/NGOs, but without duplicating either. CR services should be most closely aligned with community-based services, both clinical and non-clinical. Given the multitude of factors impacting on recovery, there is no single formula with which to predict or determine outcomes and timeframes [59]. For example, within non-acute MH services the timeframe for full client engagement would typically be for a period up to 12 months, but the overall extent of involvement, including partial or backup clinical support, would be dependent on a range of individual, social and environmental circumstances. This phase should be led by CR services but with significant involvement of CMOs/NGOs and back-up from acute and emergency services. The potential service elements include: CR teams and streams supporting both targeted and continuing care roles; specialist CR interventions; intermediate (sub-acute) stay recovery units – step-up and step-down; and links with early intervention services, GPs, housing providers, employment, education and other non-acute inpatient services.
3. Reconnection - with place and society
  The aim of this phase is to reconnect and re-establish a place in the community, and to explore opportunities for independence and social inclusion with a new sense of confidence and hope, based on the competencies developed in the previous stages. Development of a supportive daily structure is highly desirable, together with progressive utilisation and refinement of skills in the pursuit of a range of personal goals. This may necessitate graduated exposure to less structured or supported situations (e.g., independent living, community, social situations). During this phase, initial steps may be guided by CR clinicians but with CMO/NGO workers assuming greater responsibility as confidence grows in the client’s ability to be more independent. Essentially, this phase is about validating the investment of hope and developing greater levels of self-esteem and self-agency through exploration of opportunity.
  One of the advantages of CMOs/NGOs lies in their capacity to build rich and full connections with other community based groups and services. These connections may open up many satisfying and life enriching opportunities for people with enduring SMI. CMOs/NGOs may assist in the exploration of these opportunities and in the development of: stable accommodation; civic and social activities, reducing social isolation; employment opportunities; recreational and sporting activities; as well as guidance in regard to relationships and existential needs. Importantly, many CMOs/NGOs have partnerships with GPs, which, together with initial support from CR services, may ensure better access and improved mental and physical health. This phase should be led by CMO/NGO services, with the level of input from CR titrated against personal recovery needs, clinical support, risk and legal issues. As the client becomes more confident in their self-determination abilities in the community, CR services should progressively withdraw, allowing the CMOs/NGOs to assume leadership. Acute MH services would always remain available for the transfer of care and joint clinical reviews. The potential service elements include: supported accommodation (low to very high residential); low support accommodation; day centres; links with GPs; specialist employment and education services; recreational and fitness centres; and home care services.

#### Illustrative IRM scenario

**Fig. 3:**
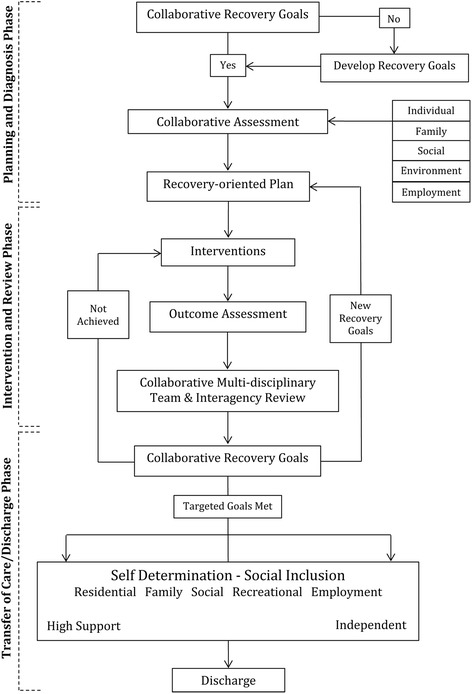
Clinical Rehabilitation (CR) processes within the IRM supporting and promoting recovery

Application of CR planning, intervention, review, transfer and evaluation processes (detailed in Table 1) within the IRM, to support and promote recovery for an individual client, are illustrated in Fig. 3. Examples of how the IRM can promote recovery for individuals with a SMI are, in most instances, complex but an illustrative scenario is provided in Table 2. Here the *remediation phase* is characterised in terms of relapse prevention and admission related decisions designed to reinstate hope, while the *restoration/reconnection phases* are illustrated via a series of recovery-focused actions in response to different concerns (e.g., about medication, treatment/intervention adherence, coping strategies to manage stress, substance misuse, family dynamics, and safety).

**Table 2:** Illustrative IRM scenario

Situation	Recovery-focussed outcome
*Remediation phase*	
**If** a client advised of an escalation in their early warning signs and… it had become evident, after review by the CR team, that coping strategies and environmental supports were not sufficiently robust to prevent a relapse.	➮ A brief admission may be considered, particularly if safety was a concern. ➮ In consultation with the acute community team, client and family, an admission plan would be developed that reassured and affirmed the client’s role in working with clinicians (e.g., in regard to identification of warning signs). ➮ The plan would also be designed to reinstate hope by building a range of coping strategies and supports. ➮ Identification of triggers &/or vulnerabilities would be central to this process. Although the initial focus would be on MH remediation, the degree of involvement of the other IRM service components would be dependent on the vulnerabilities identified by the client in the clinical review.
*Restoration/reconnection phases*	
**If** medications were a primary concern and further adjustments required.	➮ This could be managed safely in a recovery-oriented sub-acute inpatient unit, with follow-up review by the CR psychiatrist, working in conjunction with a GP.
**If**, on the other hand, adherence was a concern.	➮ Strategies could be developed by the CR clinician and, depending on the accommodation arrangements, supported by the accommodation provider. ➮ The frequency of clinical reviews would be increased to support the client and monitor effectiveness of the intervention strategies.
**If** additional coping strategies were required to manage stress in the residential or employment arena	➮ These could be developed and implemented, with the support of relevant CMOs/NGOs.
**If** issues emerged around substance misuse:	➮ The CR team would engage specialist MH services, as well as setting up risk management strategies.
**If** high levels of expressed emotion in the family were a factor.	➮ CR could develop a family intervention and education plan.
**If** the CMO/NGO indicated that there were sexual safety, antisocial or substance misuse issues in the living situation.	➮ Strategies could be developed to improve safety (before consideration of a disruptive change in location). Clinical experience would suggest that quite often a complex of vulnerabilities impacts on wellness.

*Note*: *CR* clinical rehabilitation, *CMOs*/*NGOs* community managed/non-government organisations

As an outline, this description of the IRM does not detail operational issues, such as: admission, referral and transfer processes; service hours; staff roles, competencies and training; service linkages; discharge pathways; and key performance indicators. Although these operational requirements should be guided by recovery-oriented and CR principles, other local and national factors may have an impact, including recording and reporting expectations. As with any reform, care also needs to be exercised in regard to agendas driven by vested interests and unrealistic expectations; most of all, there is a need to address the inertia within health services and to actively promote education and understanding of recovery and CR.

### Evaluation and review

#### Challenge 5

Devising implementation and evaluation strategies that enhance outcomes and facilitate review of recovery-oriented MH service models and components.

#### Evaluation goals – targets and perspectives

Evaluating the formulation, implementation and impact of specific intervention programs or MH service/practice changes can be a daunting task, especially when viewed from multiple stakeholder perspectives [91]. Expressed simply, the relevant issues are: *what aspects* of the service model are under evaluation (e.g., perceptions of practices and processes; EBI information, availability, uptake, fidelity and completion; compliance with guidelines and documentation; impact on clinical and/or personal outcomes; training and resource utilisation; and so on); from *whose perspective* (e.g., clients, carers, clinicians and/or service providers); with regard to *what timeframes* (e.g., initial impact, medium-term, ongoing); and using *what evaluation methods or strategies* (e.g., quantitative/qualitative, self-report, independent assessments, service data or other linkages).

The overriding question is: Can the chosen methods realistically address the identified evaluation goals within the required timeframes? In all likelihood, an assortment of evaluation strategies will be required, which vary in intensity and duration. Operationalising aspects of an evaluation could begin with a review of core resource materials and identified service pathways. For example, the 12 ‘clinical review’ items listed in the top right-hand corner of Table 1 could form the basis for a self-evaluation of CR processes within a particular service stream. Similarly, the flow diagram in Fig. 3, which depicts IRM processes and phases, could provide a useful starting point for auditing progress for a sample of clients and identifying service barriers (e.g., evidence in clinical records of collaborative assessments and care planning, provision of EBIs, multi-disciplinary and interagency reviews).

#### Evaluation strategies – methods and measures

Ideally, program and service evaluations should incorporate a mixture of qualitative and quantitative methods, including: reviews of available evidence; client/carer/staff structured interviews or surveys; service audits; focus groups; first-person narratives and other feedback; and assessments of recovery trajectories, short- and longer-term outcomes, and associated processes and predictors. The latter could include: *clinical recovery-focused evaluations* (e.g., symptoms, medication compliance, relapse); *personal recovery-focused evaluations* (e.g., functioning, subjective wellbeing, independence and safety, social engagement, vocational activities, quality of life, community linkages); and *service-related outcomes and evaluations* (e.g., hospital presentations, contacts with community services, engagement/referral patterns, service transitions, staff perceptions and training, policy and guideline awareness, and associated costs).

There is a growing literature on the selection of strategies and measures for assessing recovery [4, 92–94], recovery-oriented practice [65, 74, 95] and the recovery-orientation of services [71, 92, 96, 97]. In choosing a particular set of tools, it may be useful to cover a representative range of recovery domains or processes, such as the CHIME spectrum described earlier [38, 64] or the ‘broad superordinate recovery dimensions’ suggested by Whitley and Drake [98] (i.e., clinical, existential, functional, physical, and social dimensions of recovery). More generally, the capacity for client/carer self-evaluation of progress, for continuous review of recovery-oriented practices, and for reporting on key service outcomes and processes, need to become routine aspects of MH service provision, as recommended in several guidelines e.g., [31].

#### Preliminary local evaluations

While the IRM was developed as a service-wide model, it also provides an overarching framework for progressive MH service changes and EBI refinement. Like other programs [66, 68, 70], a staggered IRM introduction is probably more practical and likely to be endorsed; consequently, flexible, staged evaluation programs are also required. In our case, as described below, preliminary IRM-related evaluations focused on clinician perspectives (across the whole service) and IRM implementation within a purpose-built 20-bed, sub-acute unit [35]. More extensive evaluations are planned, covering a broader array of stakeholders and timeframes.

One staff-based method for evaluating variations in recovery-orientation is to survey clinicians pre- and post-service changes. For example, we surveyed MH clinicians recently, with the intention of conducting repeat surveys after full implementation of model of care changes. Preliminary findings (*N* = 251 clinicians, see Additional file 1) suggest that acute and community MH clinicians differ in their perceptions of the relevance of a range of recovery domains (e.g., social networks and work are perceived as less relevant domains by acute care clinicians), reflective of their likely differential contributions to the remediation and restoration phases of client recovery. Other studies have identified less positive attitudes towards recovery among inpatient providers [52, 96], suggesting that treatment setting is an important factor to consider when refining recovery-oriented care practices and training.

Intrinsic to IRM evaluation and review is the ability to respond to new opportunities as they emerge and to ongoing feedback from various stakeholders. Our initial evaluation of the implementation of an IRM within a sub-acute ISMHU [35] provided preliminary confirmation that our 6-week recovery-oriented program was acceptable, valued, and capable of contributing to enhanced functioning and an improved recovery trajectory. With respect to the interface between program goals and the sensitivity of evaluation methods, within ISMHU the various EBIs (and associated program guides) were built around and expressed in comparable terms to the MHRS domains [90], the main collaborative assessment tool used within the unit, with marked admission to discharge MHRS improvements detected [35].

## Conclusions

MH services have been the subject of many reforms but have remained largely disease-focused and paternalistic. The consumer lead recovery movement advocated for the adoption of more optimistic, recovery-oriented approaches, based on their experience that recovery was possible, despite residual symptoms [1–4]. Others have suggested that better access to treatments and psychosocial EBIs [10, 14–16, 43] is essential to improve overall MH outcomes, especially given the complexity of service and organisational reform.

Notwithstanding the merits of previous approaches, the reality is that EBIs are currently under-utilised and typically not delivered within sustainable, integrated MH systems. To assist people with SMI achieve their goals, service-wide frameworks for recovery-oriented care provision are clearly needed [36–39], together with validated intervention strategies and programs [16, 38], and workforce education programs promoting recovery-enabling competencies and positive attitudes [52, 74].

In this paper, we have drawn on relevant recovery perspectives, the psychosocial rehabilitation literature, and our own clinical and service delivery experience, to document the evolution of a broad IRM for MH services, together with associated challenges. A range of national [29, 30] and State-led initiatives [34] to improve outcomes for people with SMI, including the establishment of intermediate stay units [35], provided a unique opportunity to explore recovery-oriented models of care. Based on ICF concepts [57, 89] and CR principles [19, 77], the IRM has attempted to address the broader recovery needs of people with SMI from a health rather than a disease perspective, and to view outcomes as the interaction between the health issues, the person and their environment.

It is easy to pigeonhole new service initiatives as simply ‘good clinical practice’. The IRM was developed to facilitate access to a range of recovery-oriented EBIs, integrated across the spectrum of need. The model focuses on the fundamental factors that have been shown to promote hope, recovery, self-agency, and social inclusion. The IRM includes evidence-based CR practices and processes as a substantive component (see Table 1 and Fig. 3), which have considerable potential to help realise individual goals and aspirations. However, as recovery is often complex and non-linear (see Fig. 2), the achievement of such goals is difficult in isolation, and requires the specialised contributions of acute, non-acute and community managed/non-government organisations (CMOs/NGOs).

Service delivery models such as the IRM encourage MH services to embrace opportunities to validate hope, as a critical element for people with SMI in assuming responsibility and developing skills in self-agency and advocacy. To promote ongoing refinement of recovery-oriented service models and inform policy development, wide-ranging evaluation strategies are also critical, some aspects of which have been briefly touched on in this paper, including some preliminary IRM related evaluations.

Importantly, the three core components of the IRM revolve around and interact with the person's changing recovery needs (see Fig. 1): **remediation** of functioning (directed towards reinstating hope and a sense of possibility); collaborative **restoration** of skills and competencies; and active community **reconnection**. These core components have equally significant roles to play in promoting recovery as a process and an outcome. The ‘*remediation, restoration, and reconnection*’ refrain also provides a convenient mnemonic for the broad types of support that should be expected from recover-oriented MH services. However, the inherent strength of the IRM lies not in the capabilities of each of the contributing specialties but in the potential of the tripartite collaboration to promote and sustain hope of a life beyond mental illness that is both rich and satisfying.

## Additional file

Additional file 1: Perceived impacts on and relevance of ‘recovery domains’: Responses from a recent survey of MH clinicians (*N* = 251). (DOCX 17 kb)

## Abbreviations

CMOs/NGOs: Community managed/non-government organisations
CR: Clinical rehabilitation
EBIs: Evidence-based interventions
GP: General Practitioner
ICD-10: International classification of diseases
ICF: International classification of functioning, disability and health
IRM: Integrated recovery-oriented model
ISMHU: Intermediate stay mental health unit
MH: Mental health
MHRS: Mental health recovery star
NSW: New South Wales
SMI: Serious mental illness
WHO: World health organisation
