# Supplementary material for: An Integrated Recovery-oriented Model (IRM) for mental health services: evolution and challenges
Source: BMC Psychiatry. 2017 Jan 17;17:22. doi: 10.1186/s12888-016-1164-3 (PMC5240195; doi:10.1186/s12888-016-1164-3)
Supplement: Additional file 1: — Perceived impacts on and relevance of ‘recovery domains’: Responses from a recent survey of MH clinicians (N = 251). (DOCX 17 kb) [file 12888_2016_1164_MOESM1_ESM.docx]

**Supplementary Document 1** (*December, 2016*):

**Perceived impacts on and relevance of ‘recovery domains’: Responses from a recent survey of MH clinicians (*N* = 251)**

| **Recovery domain** | **Perceived impact of treatment practices:** | **Perceived importance for care planning**  **within your unit/service:** | | |
| --- | --- | --- | --- | --- |
|  | **All clinicians**  **(N = 251)**  Mean (SD) | **All clinicians**  **(N = 251)**  Mean (SD) | **Acute inpatient MH units (N = 89)**  Mean (SD) | **Community**  **MH (N = 162)**  Mean (SD) |
| **Managing Mental Health** (managing symptoms and encouraging self-reliance) | 3.72 (0.95) | 4.13 (0.91) | 3.79 (0.98) | 4.31 (0.82)*** |
| **Living Skills** (ability to live independently, shop, cook, clean, budget) | 3.43 (1.06) | 3.41 (1.15) | 3.07 (1.14) | 3.58 (1.12)** |
| **Trust and Hope** (having a sense of hope, and trusting in self and others) | 3.36 (1.09) | 3.57 (1.13) | 3.28 (1.15) | 3.72 (1.10)* |
| **Identity and Self Esteem** (liking who they are, satisfying sense of self) | 3.31 (1.06) | 3.50 (1.14) | 3.14 (1.06) | 3.69 (1.14)** |
| **Social Networks** (encouraging participation and community activities) | 3.27 (1.09) | 3.49 (1.14) | 3.06 (1.20) | 3.72 (1.05)*** |
| **Self-care and Physical Health** (managing physical health and self-care) | 3.23 (1.01) | 3.62 (1.03) | 3.55 (1.01) | 3.66 (1.04) |
| **Relationships** (developing closeness with family, friends or partner) | 3.21 (1.03) | 3.51 (1.11) | 3.26 (1.08) | 3.64 (1.10) |
| **Responsibilities** (self-managing daily responsibilities - i.e., bills, neighbours) | 3.21 (1.03) | 3.44 (1.08) | 3.11 (1.16) | 3.61 (1.01)** |
| **Addictive Behaviour** (developing coping strategies to reduce potential harm) | 3.13 (1.05) | 3.54 (1.09) | 3.33 (0.98) | 3.65 (1.13) |
| **Work** (full or part-time work, studying or volunteering) | 3.12 (1.06) | 3.24 (1.20) | 2.59 (1.07) | 3.57 (1.13)*** |
| **Overall** (average rating) | 3.30 (0.89) | 3.54 (0.88) | 3.21 (0.93) | 3.71 (0.81)*** |
| ***Note***. Surveys of MH clinicians (Nursing, 45.4%; Allied Health, 40.2%; Medical/Other, 14.3%) were conducted prior to proposed model of care changes. Some questions were framed in terms of the ten Mental Health Recovery Star (MHRS) domains: “*How much impact (for clients) do you feel our current treatment practices are likely to have on each of these recovery domains?*”; and “*In developing care plans for clients, how much importance does your unit/service place on each of the following recovery domains?*”. Five-point rating scales were used (1: None; 2: Some; 3: Moderate; 4: Considerable; 5: High). Statistical comparisons between service location/type based sub-groups controlled for clinicians’ professional background.  ***Conclusion***. The ‘managing mental health’ domain received consistently higher ratings, while the ‘work’ domain received the lowest ratings, particularly among clinicians from acute inpatient MH units. With respect to perceived importance for care planning, ratings for the recovery domains were substantially higher from community MH clinicians (0.50 points on average), with statistically significant differences on seven of the ten domains: * *p* < .05; ** *p* < .01; *** *p* < .001. | | | | |
